# Animal Use and Lessons Learned in the U.S. High Production Volume Chemicals Challenge Program

**DOI:** 10.1289/ehp.1104666

**Published:** 2012-10-02

**Authors:** Patricia L. Bishop, Joseph R. Manuppello, Catherine E. Willett, Jessica T. Sandler

**Affiliations:** People for the Ethical Treatment of Animals, Regulatory Testing Division, Norfolk, Virginia, USA

**Keywords:** alternative methods, animal testing, chemical risk assessment, High Production Volume Chemicals Challenge Program, high production volume, PETA, REACh. *Environ Health Perspect* 120:1631–1639 (2012)

## Abstract

Background: Launched by the U.S. Environmental Protection Agency (EPA) in 1998, the High Production Volume (HPV) Challenge Program was developed to address the perceived gap in basic hazard information for the 2,800 chemicals produced or imported into the United States in quantities of ≥ 1 million pounds per year. Health and environmental effects data obtained from either existing information or through new vertebrate animal testing were voluntarily submitted by chemical companies (sponsors) to the U.S. EPA. Despite the potential for extensive animal testing, animal welfare guidelines were not provided until after the start of the program.

Objectives: We evaluated compliance with the animal welfare principles that arose from an agreement reached between the U.S. EPA and animal protection organizations and tracked the HPV program’s use of animals for testing.

Discussion: Under a worst-case scenario, the HPV program had the potential to consume 3.5 million animals in new testing. After application of animal-saving measures, approximately 127,000 were actually used. Categorization of chemicals based on similar structure–activity and application of read-across, along with use of existing test data, were the most effective means of reducing animal testing. However, animal-saving measures were inconsistently used by both sponsors and the U.S. EPA.

Conclusions: Lessons learned from the HPV program can be applied to future programs to minimize animal testing and promote more human-relevant chemical risk assessment.

Launched in October 1998 as part of the Chemical Right-to-Know initiative [U.S. Environmental Protection Agency (EPA) 1998b], the High Production Volume (HPV) Chemicals Challenge Program (HPV program) was developed by the U.S. EPA in concert with the Environmental Defense Fund (EDF; a nongovernmental environmental advocacy group), the American Petroleum Institute (API), and the Chemical Manufacturer’s Association [now the American Chemistry Council (ACC)]. The HPV program focused on chemicals produced in or imported into the United States in annual quantities of ≥ 1 million pounds, which in 1998 amounted to approximately 2,800 substances. The stated goals of the HPV program were to collect health and environmental effects data and provide the public with basic hazard information on these chemicals that would allow individuals to actively participate in environmental decision making (U.S. EPA 2011e).

Chemical companies were encouraged to volunteer for the HPV program or face regulation under the Toxic Substances Control Act (TSCA 1976). For each chemical that was sponsored, the U.S. EPA requested all the information specified in the Organisation for Economic Co-Operation and Development (OECD) HPV Screening Information Data Set (SIDS) (OECD 2012b). The OECD, as one of its functions, sets international standards and publishes validated methodologies for chemical safety testing. Developed for the OECD HPV Chemicals Programme, SIDS consists of physicochemical information and data on environmental fate/pathways, ecotoxicity, and mammalian toxicity (U.S. EPA 2010a).

## Animal Testing and Introduction of Animal-Saving Measures

To satisfy SIDS ecotoxicity and human health effects data requirements that relied on animal data (end points), a chemical sponsor could either submit existing animal test results or conduct new animal tests. Considering the 2,800 chemicals identified and the amount of test data sought, the HPV program had the potential to consume millions of animals in new testing efforts, yet there had been no participation by animal protection organizations (APOs) in its planning. Subsequent critiques of the HPV program by People for the Ethical Treatment of Animals (PETA); the Physicians Committee for Responsible Medicine; other nongovernmental organizations including the Doris Day Animal League, the American Anti-Vivisection Society, and the Medical Research Modernization Committee; and the public eventually led to an agreement with the White House and the U.S. EPA to include a number of animal protection measures in the HPV program (e.g., Hess 1999; Lazaroff 1999; PETA 1999). This agreement set a precedent in the government’s incorporation of animal welfare concerns into federal testing requirements.

The agreement, issued in the form of a letter from the U.S. EPA (Wayland 1999) that outlined the new guidelines for animal use, was sent to all participating companies. HPV program participants were directed to *a*) not perform an animal test when a validated non-animal method was reasonably and practically available; *b*) use existing, scientifically adequate data to the maximum extent, including information from international chemical databases; *c*) use *in vitro* genetic toxicity testing unless known chemical properties precluded its use; *d*) conduct a thoughtful, qualitative analysis, including consideration of a substance’s physicochemical properties; *e*) apply a weight-of-evidence (WoE) approach whenever possible and forgo conducting certain tests if appropriate; and *f* ) maximize grouping of related chemicals into categories based on structure–activity relationships (SARs). In addition, sponsors were told not to develop subchronic or reproductive toxicity data for closed system intermediates (CSIs; chemicals that are used to produce another chemical and that are handled in ways that result in a low possibility of exposure), and to consider whether any additional information obtained through new testing would be useful or relevant for substances generally recognized as safe by the Food and Drug Administration (FDA). Finally, the U.S. EPA agreed to incorporate these elements into future HPV test rules (Wayland 1999).

Thus, in theory, several means existed at the start of the HPV program to satisfy the health and environmental effects end points requiring animal data while also meeting the goal of minimal animal use in testing. These animal-saving measures are summarized in Table 1.

**Table 1:** Animal-saving measures available to satisfy health and environmental effects end points and minimize animal testing in the HPV program

Methodology	Animal-saving measures
Existing data	Submitting existing test results for specific SIDS end point
Read-acrossa	Grouping chemicals based on SARs and using read-across from tested chemicals to evaluate analogous untested chemicals
Practical considerationsb	Obviating tests based on physicochemical or biological properties, exposure route, or use; observed effects from previous non-SIDS tests; reproductive toxicity satisfied by lack of observed effects on reproductive organs in a repeat dose test of ≥ 90 days plus negative findings from an existing developmental toxicity study; WoE; GRAS substances; and other relevant information
Non-animal methods	In vitro methods for genetic toxicity and quantitative (Q)SAR computer programs, such as ECOSAR, which estimates toxicity to fish, invertebrates, and algae
CSIs	Appropriately classifying chemicals as CSIs, and thereby avoiding the need for repeat dose toxicity and reproductive toxicity tests, which were not required for CSIs
Abbreviations: CSIs, closed system intermediates; ECOSAR, Ecological Structure Activity Relationships (U.S. EPA 2011c); GRAS, generally recognized as safe; (Q)SAR, (quantitative) SAR; SAR, structure–activity relationship. aProcess by which end point information for one chemical is used to predict the same end point for another chemical based on similarities in their chemical structure or functionality. bThe U.S. EPA’s letter (Wayland 1999) urged sponsors to conduct a “thoughtful, qualitative analysis”; we termed the animal-saving measures covered under this umbrella “practical considerations.”		

The HPV test battery included a total of six vertebrate animal–based end points: five for human health effects (acute toxicity to mammals, repeated dose toxicity, reproductive toxicity, developmental toxicity, and genetic toxicity) and one for environmental effects (acute toxicity to fish) (U.S. EPA 2000). Table 2 summarizes the vertebrate animal tests available to satisfy these end points, identification numbers for the appropriate OECD test guideline (TG; OECD 2012a), and the number of animals associated with each test. In general, the U.S. EPA (2000) recommended that combined protocols [TG 421 (Reproduction/Developmental Toxicity Screening Test) or TG 422 (Combined Repeated-Dose Toxicity Study with the Reproduction/Developmental Toxicity Screening Test)] be used to screen multiple end points; these tests require approximately half the number of animals called for in the separate developmental toxicity test (TG 414; Prenatal Development Toxicity Study) or reproductive toxicity test (TG 415; One-Generation Reproduction Toxicity Study). The genetic toxicity end point includes gene mutation and chromosomal aberration/damage (CAD). The U.S. EPA recommended the use of *in vitro* assays for gene mutation. For CAD, sponsors could use either the *in vitro* (TG 473; In Vitro Mammalian Chromosome Aberration Test) or the *in vivo* (TG 474; Mammalian Erythrocyte Micronucleus Test) method, but they were asked to provide a rationale for proposing the animal test instead of the *in vitro* assay. One chemical tested for all animal test end points would require, on average, 60 fish and up to 2,480 mammals (Table 2) if separate repeated-dose tests, developmental and reproductive toxicity tests, and the *in vivo* CAD test were used.

**Table 2:** Animal tests used in the HPV Chemicals Challenge Program animal tests, OECD test guideline (TG) number, and numbers of animals associated with each test.

Test	OECD TGa	Animals used [n or median (range)]
Acute toxicity, fishb	203	60
Acute toxicity, mammal
Oral, up-and-down methodb	425	10 (6–15)
Acute oralc	401	23 (20–25)
Acute inhalationb	403	23 (20–25)
Oral, toxic class method	423	9 (6–12)
Repeated dose toxicity
28-Day oralb	407	40
28-Day inhalation	412	53 (40–65)
90-Day oral	408	80
90-Day inhalation	413	80
Developmental toxicity
Prenatal developmental toxicity	414	1,160
Reproductive toxicity
One-generation reproduction	415	1,160
Combined protocols
Reproductive/developmental toxicity screeningb	421	580
Repeated dose/reproductive/developmental toxicity screeningb	422	580
Genetic toxicityd
Mammalian erythrocyte micronucleus CAD	474	50
CAD, chromosomal aberration/damage. aTG 203 (OECD 1992); all other TGs (OECD 2012a). bTest recommended in HPV program guidance (U.S. EPA 2000). cThis test was deleted from the manual of accepted OECD test guidelines in 2002; however, it was included in a few early proposals. dHPV program guidance for the genetic toxicity CAD end point was the in vitro TG 473.				

## Objectives

As of January 2010, sponsors had submitted 428 test plans to the U.S. EPA to address 1,420 of the original 2,800 HPV chemicals, most of which were grouped in categories containing two or more related chemicals. The plans present existing data that satisfy some or all SIDS requirements, and propose new tests to fill any perceived data gaps. Here, we review these plans and accompanying documents, focusing primarily on compliance with the principles contained in the animal welfare guidance and on the resulting impact of proposed and actual tests on animal use. We also discuss which animal-saving measures were most effective for reducing the number of animals used.

## Methods

We reviewed all publicly available test plans, the U.S. EPA comments on those test plans, and test plan revisions available online in “Robust Summaries and Test Plans” (U.S. EPA 2012d). Although the U.S. EPA commented on 413 of the 428 test plans submitted, sponsors subsequently revised only 330. We based our analysis on original test plans, the U.S. EPA comments, and test plan revisions available from the HPV program (U.S. EPA 2012d) as of January 2010.

For each health and environmental effect end point potentially requiring animal test data, we determined whether new animal testing was proposed in original test plans and if not, which of the animal-saving measures listed in Table 1 was used to satisfy the end point. If new animal testing was proposed, we noted the OECD TG to be followed; if no TG was specified, we assumed that the tests listed in program guidance (U.S. EPA 2000) were to be used [i.e., TG 203 (OECD 1992) for acute toxicity to fish, TG 425 or TG 403 for acute mammalian toxicity, TG 421 for developmental and/or reproductive toxicity, TG 422 for repeated dose along with developmental and/or reproductive toxicity, and TG 473 for CAD] (Table 2). We tallied the final number of each type of animal test or animal-saving measure used by recording how each end point was addressed in the most recent document posted in “Robust Summaries and Test Plans” (U.S. EPA 2012d), that is, either: *a*) the original test plan, if no U.S. EPA comments on that test plan or subsequent revisions were posted; *b*) the U.S. EPA comments on the original test plan if no subsequent revisions were posted; or *c*) the latest revision posted in response to U.S. EPA comments.

After reviewing original test plans, the U.S. EPA generally provided a response for each animal test proposed by sponsors, indicating *a*) that the test was accepted as proposed; *b*) that the proposed test was unnecessary; or *c*) that the proposed test could be replaced with a different test requiring fewer or no animals. The U.S. EPA also recommended additional tests in cases where it did not accept one or more animal-saving measures proposed by sponsors to satisfy required end points. In revised test plans submitted by sponsors, we noted whether or not they agreed to make the changes recommended by the U.S. EPA. If sponsors had not responded, we counted the tests recommended in the U.S. EPA comments.

In some cases involving complex mixtures and process streams, chemical companies proposed new testing for related non-HPV substances rather than for the sponsored chemicals themselves. We included the tests used for these substances in our animal test totals, and end points for the sponsored chemicals were counted as satisfied by read-across, a process by which end point information for one chemical is used to predict the same end point for another chemical based on similarities in their chemical structure or functionality. In several cases for which sponsorship of chemicals was withdrawn due to overlap with international regulatory programs, we considered the end points addressed by existing data. In some situations the U.S. EPA accepted or rejected proposed animal-savings measures based on the sponsor meeting certain conditions, such as supplying study details in a robust summary or locating additional studies. If sponsors did not make revisions to test plans or if it was unclear as to what was actually done, we judged whether those conditions were likely to be met and how the end points were eventually satisfied.

## Discussion

*Analysis of animal use.* Based on the median or standard number of animals used per test (Table 2), approximately 3.5 million animals would have been required to conduct a complete OECD SIDS battery on the 1,420 chemicals sponsored in the HPV program, using separate tests for each end point. This estimate would be reduced to about 994,000 animals if combined protocols were used for repeated dose/reproductive/developmental toxicity end points instead of separate tests. Because animal-saving measures were used, the actual number of animals killed was substantially reduced, but still amounted to nearly 127,000.

**Figure 1:**
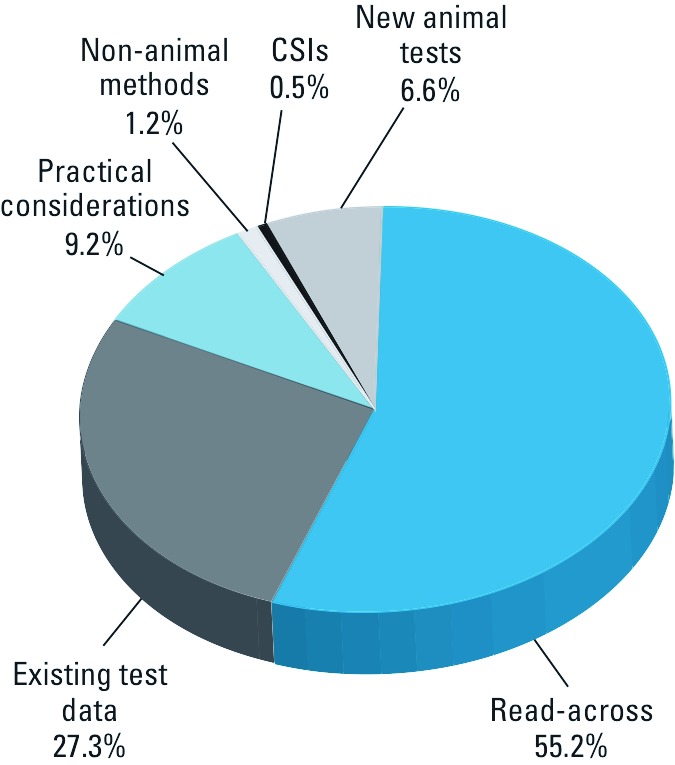
Percentages of end points requiring animal data satisfied by animal-saving measures or new animal tests for the 1,420 sponsored HPV chemicals. For some chemicals, animal tests were performed on non-HPV chemicals or mixtures of chemicals and the results of these tests were used to generate read-across for the HPV chemical; in these cases, we counted both the animal test and read-across toward fulfilling the end point.

In Figure 1, we summarize the extent to which the animal-saving measures listed in Table 1 together with new animal tests were used to satisfy all of the health and environmental effects end points potentially requiring animal test data for the 1,420 sponsored chemicals. Placing chemicals into categories and applying read-across from animal tests already conducted or proposed for analogous chemicals satisfied 55% of these end points. Submittal of existing test data also reduced animal use considerably, satisfying 27% of the end points. Such extensive availability of data for analogous chemicals and existing test results contrasts sharply with findings in two reports that were largely responsible for the creation of the HPV program. In *Toxic Ignorance*, the EDF (1997) stated that

Today, even the most basic toxicity testing results cannot be found in the public record for nearly 75% of the top volume chemicals in commercial use.

Likewise, the U.S. EPA reported a paucity of data in its follow-up publication *Chemical Hazard Data Availability Study: What Do We Really Know About the Safety of High Production Volume Chemicals?* (U.S. EPA 1998a). However, the Physicians Committee for Responsible Medicine (1998) found that the EDF and U.S. EPA had overlooked many databases containing toxicological data drawn from a wide variety of sources; these databases were later described in the U.S. EPA’s letter (Wayland 1999) as being available for use by sponsors.

Practical considerations—mainly cases in which sponsors determined a chemical to be ineligible for testing based on its physicochemical properties—saved animals as well, avoiding testing for 9.2% of the end points (Figure 1). This approach was exemplified by butyllithium, a chemical described by its sponsor, the FMC Corporation, Lithium Division (FMC 2002), as extremely reactive with air, moisture, and animal tissues. The FMC further concluded that exposure of butyllithium to test animals would be cruel and would not generate meaningful data because the test animals would most likely have to be killed for humane reasons long before the end of the study (FMC 2002). In the U.S. EPA’s comments on the test plan, Hernandez (2002a) agreed with the unsuitability of butyllithium for SIDS testing, stating that

Owing to the highly reactive nature of this chemical when in contact with air or water, it is not feasible to perform physicochemical, environmental fate, mammalian or ecotoxicological tests.

Non-animal methods, such as the *in vitro* CAD test, were used sparingly, accounting for only 1.2% of end points. A chemical’s status as a CSI satisfied requirements for < 1% of end points (Figure 1).

Data requirements for the remaining 6.6% of end points were met by conducting new animal tests. Of the 334 tests proposed by sponsors in original test plans, the U.S. EPA accepted 223, rejected 49 as not needed, and substituted 62 tests that used fewer or no animals (Table 3). Most of the substitutions involved replacing *in vivo* CAD tests with *in vitro* assays and using combined protocols (TG 421 or TG 422) to evaluate reproductive and/or developmental toxicity, with or without repeated dose toxicity. The U.S. EPA’s recommendations regarding elimination of tests and replacement with less animal–intensive tests would have resulted in nearly 36,000 fewer animals used than originally proposed (Table 3).

**Table 3:** The U.S. EPA’s recommendations regarding animal tests originally proposed by sponsors (i.e., test accepted, test not needed, or test substituted with one using fewer or no animals) and number of animals potentially saved.

	Original sponsor plans (n)				U.S. EPA suggested (n)	
Test	Proposed tests	Test accepted	Test not needed	Animals saved^a^	Tests using fewer/no animals	Animals saved^b^
Acute toxicity, fish	87	77	10	660	NAc	—
Acute toxicity, mammal	26	7	17	170	2	26
Repeated dose toxicity	23	2	4	160	17	892
Developmental toxicity	29	1	4	4,640	24	15,080d
Reproductive toxicity	11	3	2	2,320	6	4,640d
Combined protocols	129	118e	11	6,380	NAc	—
CAD (in vivo)	29	15	1	50	13	650
Total	334	223	49	14,320	62	21,288
aNumber of animals saved by eliminating tests, based on standard or median number of animals per test shown in Table 2. bNumber of animals saved by using tests with fewer/no animals, based on standard or median number of animals per test as shown in Table 2. cNot applicable (i.e., there was no substitute test for combined protocols, which already used fewer animals, or for the acute toxicity to fish test). dThe U.S. EPA recommended 22 combined protocols in place of either reproductive or developmental toxicity and four combined protocols in place of both reproductive and developmental toxicity. The animal savings by end point was calculated as follows: developmental toxicity = 20 × (1,160 – 580) + 4 × [(2,320 – 580)/2] = 15,080; reproductive toxicity = 2 × (1,160 – 580) + 4 × [(2,320 – 580)/2] = 4,640. eThe U.S. EPA made no comment for 2 tests, which we assumed to have been accepted.												

Due to the voluntary nature of the program and stated commitments to certain product stewardship efforts, sponsors did not always comply with the U.S. EPA’s recommendations to eliminate tests or to substitute tests using fewer or no animals. Sponsors went on to conduct 12 of the 49 tests that the U.S. EPA determined were not needed, including 5 fish toxicity tests using 300 fish, 4 acute mammalian toxicity tests using about 60 rodents, and 3 combined protocol tests using 1,740 animals. Sponsors also did not perform 21 of the 62 less animal–intensive tests recommended by the U.S. EPA and, instead, conducted the tests originally proposed [i.e., 5 repeated-dose tests (200 animals), 8 developmental tests using TG 414 (4,640 more animals), 3 reproductive tests using TG 415 (1,740 more animals), and 5 *in vivo* CAD tests (250 animals). Thus, almost 9,000 more animals were used in the HPV program as a result of sponsors’ failure to follow the U.S. EPA’s recommendations. Of these, 6,380 could have been spared if the sponsors had used combined protocols instead of separate reproductive or developmental toxicity tests.

During its review of test plans, the U.S. EPA also recommended that 154 additional tests be conducted, which, based on the median or standard number of animals per test in Table 2, would have used 57,000 animals. Of these 154 tests, sponsors agreed to conduct 75 (44 combined protocols, 29 acute fish, 1 developmental, and 1 *in vivo* CAD), using about 28,500 animals, and declined to conduct 79 (46 combined protocols, 29 acute fish, 3 acute mammal, and 1 repeated dose), saving about 28,500 animals. For reasons not necessarily related to the HPV program, sponsors also added 43 tests (13 acute fish, 9 combined protocols, 8 repeated dose, 7 *in vivo* CAD, 3 acute mammal, 2 reproductive, and 1 developmental) after U.S. EPA review, using approximately 10,200 animals.

We estimated the net effect of the U.S. EPA review and sponsor response on animal numbers by comparing the number of animals required for the original 334 test plans submitted by sponsors to the number required according to the most recent documents available in the the U.S. EPA’s Robust Summaries and Test Plans (U.S. EPA 2012d) (Table 4). Although the total number of tests increased from 334 to 349 after the U.S. EPA review, the number of animals used decreased by about 3,000 to 126,460. This decrease was due not only to the U.S. EPA’s recommendations to eliminate tests or to substitute tests with fewer or no animals but also to sponsors’ declining to conduct additional testing recommended by the U.S. EPA and, in a few cases, to sponsors’ decisions to drop tests they had originally proposed. If all of the U.S. EPA’s recommendations had been followed, there would have been a net increase of 21,000 animals used because, while the U.S. EPA’s rejection of tests as unnecessary and its recommendations to conduct tests using fewer or no animals would have reduced animal numbers by about 36,000 as noted above, the 154 additional tests requested would have increased animal usage by about 57,000.

**Table 4:** Initial number of tests proposed and animals required versus final number of tests in most recent program documents (revised test plan or U.S. EPA comments) and animals required.

	Initial (n)		Final (n)	
Test	Proposed tests	Animals requireda	Tests	Animals requireda
Acute toxicity, fish	87	5,220	111	6,660
Acute toxicity, mammal	26	440	16	185
Repeated dose toxicity	23	1,172	15	755
Developmental toxicity	29	33,640	11	12,200
Reproductive toxicity	11	12,760	8	9,280
Combined protocols	129	74,820	166	96,280
CAD (in vivo)	29	1,450	22	1,100
Total	334	129,502	349	126,460
aNumber of animals based on standard or median number of animals per test as shown in Table 2.								

*Compliance with the animal welfare agreement.* An early review of the HPV program by the Physicians Committee for Responsible Medicine (Cardello 2001) documented serious flaws in test plans submitted by sponsors, including *a*) failure to report existing hazard information and to group structurally or toxicologically similar chemicals; *b*) proposed animal tests that were beyond the scope of the HPV program; and *c*) lack of enforcement by the U.S. EPA of agreed-upon animal welfare principles. A subsequent evaluation of the HPV program by Nicholson et al. (2004) showed that many of the same problems reported by Cardello (2001) still existed. In addition, they found that testing was proposed for chemicals with known toxicities and for irrelevant end points when the primary hazard was high and well known, and that testing *in vivo* was proposed when valid *in vitro* methods were available.

In our analysis, we found inconsistencies both in the U.S. EPA’s treatment of the information submitted by sponsors and in sponsors’ adherence to the animal welfare guidelines. For example, similar to the case made by the FMC for butyllithium, the sponsor for benzene phosphinic acid (phenylphosphinic acid) proposed no additional mammalian toxicology testing because existing animal data showed that administration by oral gavage causes gastrointestinal tract bleeding, necrosis, and occasionally perforation, and the corrosive effects of this substance had already been demonstrated as the basis for its toxicity (BPD/BPA Coalition 2003). In its review, the U.S. EPA (Hernandez 2004) cited a 14-day repeated dose study (dietary exposure) conducted in 1981 (Haskell Laboratories 1982) that found lower doses of benzene phosphinic acid did not appear to cause animals distress and recommended a combined repeated dose/reproductive/developmental toxicity test (TG 422) be done despite the animal welfare concerns of the sponsor. In the final robust summaries report (a robust summary describes the objectives, methods, results, and conclusions of a full study in enough detail to allow a technically qualified person to make an independent assessment of that study) for benzene phosphinic acid, the BPD/BPA Coalition (2004) indicated that the sponsor conducted a new oral repeated dose study (28-day study in rodents using TG 407) in 2003, which showed essentially the same no observed adverse effect level result (779 mg/kg males; 859 mg/kg females) as the 1981 study (863 mg/kg). To satisfy the reproductive/developmental toxicity end points, however, the sponsor used data from a 1996 test on a similar substance, toldimfos.

Even with recommendations by the U.S. EPA to use existing information, some chemical sponsors still failed to summarize all available data and instead proposed animal tests. For its fuel oils category, the ACC (2001) proposed evaluation of acute aquatic toxicity with two fish tests (using a total of 120 fish), despite already possessing data on this end point for similar products. In addition, the ACC acknowledged in its test plan that these substances consist of neutral organic hydrocarbons, whose toxic mode of action is well understood to be nonpolar narcosis. When these fish tests were conducted on two representative oils in 2004 (ExxonMobil Biomedical Sciences, unpublished data), the median lethal concentration and median lethal level were within the range of acute fish toxicity data already reported by the ACC for this category in its original test plan (ACC 2001). Moreover, in the final robust summaries for this category, the ACC (2005) cited two 1998 fish studies (Targia ME, Freeman JJ, unpublished data) performed with No. 2 fuel oil that were apparently overlooked in 2001 when the original test plan was prepared.

Contrary to its own guidance to “… conduct a thoughtful, qualitative analysis rather than use a rote checklist approach” (Wayland 1999), the U.S. EPA sometimes applied a more narrow definition of program requirements when it rejected existing toxicity and exposure data and instead recommended new animal tests. This was evident in the U.S. EPA’s call for an acute fish test for the mononitrile category (Hernandez 2003), despite the sponsor’s determination that no additional testing was needed based on the combined evaluation of data from several existing fish and invertebrate studies (Brooke et al. 1984; Dupont Haskell Laboratory, unpublished data), application of the predictive computer program ECOSAR (U.S. EPA 2011c), the physicochemical characteristics of the compounds, and the limited potential for meaningful aquatic exposures (Dupont 2002, 2004). Another example is the U.S. EPA’s treatment of the ionone derivatives category, substances which naturally occur in plants containing β-carotene. The Flavor and Fragrance High Production Volume Consortia (FFHPVC), which sponsored this category, cited studies showing that human exposure is more likely via consumption of fruits and vegetables than by consumption of products flavored with these substances (Stofberg and Grundschober 1987; Stofberg and Kirschman 1985) and noted that ionone derivatives are recognized by the FDA as being generally recognized as safe for their intended use in food (FFHPVC 2002). Based on these factors and existing data from studies already conducted, the FFHPVC proposed no new animal tests, yet the U.S. EPA recommended a new developmental toxicity test that uses > 1,000 animals (Hernandez 2002b). Rather than conduct the new test, the sponsor provided in its revised test plan (FFHPVC 2004) a more comprehensive analysis of data from a 1986 developmental study on hamsters (Willhite 1986) that had already been cited in the original test plan.

Grouping related chemicals into categories offers a means not only for reducing the number of new animal tests required but also for providing a contextual basis from which to evaluate toxicity. Of the 428 original test plans reviewed by the U.S. EPA, 125 are for categories of related chemicals accounting for 1,117 of the 1,420 sponsored chemicals. Yet, additional opportunities to group related chemicals into categories were missed, resulting in duplicative and inefficient testing strategies. For example, tris(nonylphenol) phosphite, sponsored by the Phosphite Producers HPV Consortium, could have been assessed in the context of a larger group of phenyl-phosphorus antioxidant stabilizers, and *p*-cumylphenol, sponsored by General Electric, could have been included in a larger substituted or alkylphenol category (Cardello 2001).

Use of non-animal methods to reduce animal testing was not fully exploited despite the October 1999 guidance letter (Wayland 1999) clearly stating that validated non-animal methods should be used whenever possible. The 96 *in vitro* CAD (TG 473) tests proposed in the most recent test plans or U.S. EPA comments did spare the lives of 4,800 animals, but 29 *in vivo* CAD (TG 474) tests were also proposed, with only six sponsors submitting the required justification for using this assay. The U.S. EPA rejected one of the *in vivo* tests entirely and recommended use of the *in vitro* test instead in 13 cases (Table 3); however, 22 *in vivo* CAD tests still were performed, killing 1,100 animals (Table 4).

Another non-animal method with the potential to reduce animal use was the ECOSAR computer program (U.S. EPA 2011c), which predicts aquatic toxicity based on SARs. Although the U.S. EPA described this (quantitative) SAR method as providing screening-level characterization of ecotoxicity end points, including acute toxicity to fish, it still generally required fish test data from an analog to be summarized whenever ECOSAR was used (U.S. EPA 2010d), severely limiting the potential of the model to reduce fish use. This limitation appears to contradict the U.S. EPA’s own use of ECOSAR estimates (U.S. EPA 2011c):

The U.S. EPA Office of Pollution Prevention and Toxics uses SARs to predict the aquatic toxicity of new industrial chemicals in the absence of test data. … Environmental assessors, chemical manufacturers, chemical suppliers, and other regulatory agencies have used ECOSAR to develop quantitative screening level toxicity profiles.

Sponsors substituted ECOSAR data in place of animal tests only 29 times in the absence of analog test data, and the U.S. EPA rejected 11 of the proposed substitutions.

The U.S. EPA also recognized that chemicals with high *n*-octanol/water partition coefficients (*K*_ow_) are less likely to be toxic to fish; thus, in its program guidance, the U.S. EPA recommended that a chronic toxicity to *Daphnia* test be conducted—instead of acute toxicity to fish—for chemicals with a log *K*_ow_ ≥ 4.2 (U.S. EPA 2000). Surprisingly, sponsors proposed 18 new fish tests for chemicals that met the *K*_ow_ criteria for use of *Daphnia* data, and the U.S. EPA accepted 16 of these test proposals, although sponsors subsequently dropped 6 of the proposed fish tests in test plan revisions.

Although a substance’s solubility in water should have been a primary consideration in determining whether to test for aquatic toxicity, fish testing was nevertheless conducted on substances with very low solubility. For example, in the Pine Chemicals Association’s (PCA) test plan for rosin (a naturally occurring substance from pine trees that is used in chewing gum, printing ink, adhesives, and coatings) and rosin salts (used in paper products, soaps, and detergents), the PCA (2001) acknowledged that rosin was essentially insoluble in water. Yet, it went on to conduct acute toxicity tests on fish, *Daphnia*, and algae, the results of which showed that none of the compounds in this category were toxic to aquatic organisms (PCA 2004).

Choosing one of the combined protocols (TG 421 or TG 422), each of which uses 580 animals (Table 2) to screen for reproductive and developmental toxicities, had the potential to save many animals, compared with conducting separate tests for these end points, which would require 1,160 animals/test. Sponsors initially proposed 129 combined protocols, potentially saving 75,000–225,000 animals, depending on whether the combined test replaced one or both of the separate tests. The U.S. EPA recommended TG 421 or TG 422 tests in place of 24 proposed TG 414 tests and 6 proposed TG 415 tests (Table 3), but only 17 of these recommendations were accepted. Nevertheless, combined tests had a significant impact on reducing the number of animals used in testing HPV chemicals.

Sponsors cited a substance’s physical, chemical, or biological properties as a reason for precluding animal testing for 561 end points. However, some sponsors still proposed animal tests, even when a chemical’s properties rendered the results of these tests meaningless. In the API’s initial test plan for the Petroleum Gases Category, its Petroleum HPV Testing Group (PHTG) proposed separate acute mammalian, repeated dose, reproductive, and developmental toxicity tests on each of the individual gases ethane, butane, propane, and isobutane, even though these gases are explosive at concentrations below those at which health effects are observed and have been shown to act primarily as simple asphyxiants (Nicholson et al. 2004). After receiving comments from APOs and the U.S. EPA, the PHTG reconsidered its testing proposal and eliminated from its revised test plan all acute mammalian tests and all separate reproductive and developmental toxicity tests on individual gases (Twerdok 2001). However, the PHTG still conducted combined protocol tests on the four individual gases, which showed no or very minor health effects (PHTG 2009). Interestingly, the PHTG’s original plan called for no testing of methane because of its physicochemical properties. Despite the U.S. EPA’s disagreement with this finding, the PHTG refused to change its position (PHTG 2001), maintaining that

The physical properties and ubiquitous presence of methane in the environment (including being a metabolic product of intestinal bacteria in humans) make health effects testing on methane unnecessary.

Notwithstanding its own guidance that participants need not develop certain data for chemicals that were solely CSIs, the U.S. EPA rejected 33 of 74 sponsor claims that testing for repeated dose and reproductive toxicity was not needed based on a chemical’s classification as a CSI. The agency often failed to give specific reasons for rejecting these claims, only listing the CSI requirements and stating that the information provided was inadequate to support them.

The U.S. EPA agreed to consider a lack of effects on the reproductive organs observed in a 90-day repeated dose toxicity test as a means of satisfying the reproductive toxicity end point when a developmental toxicity study was also available, as provided for in OECD SIDS guidance (U.S. EPA 2010a). Although the agency did reject two proposed reproductive toxicity (TG 415) tests on this basis, it went on to recommend new testing on 14 chemicals for which sponsors had submitted lack-of-effects data because, in most of these cases, the data submitted failed to fully meet the U.S. EPA’s established criteria for waiving the reproductive toxicity test (U.S. EPA 2010a).

The U.S. EPA seemed willing to accept WoE as a reason for not testing, as stated in its letter (Wayland 1999),

Participants may conclude that there is sufficient data, given the totality of what is known about a chemical, including human experience, that certain end points need not be tested.

However, for the 76 cases in which sponsors provided WoE arguments, the U.S. EPA rejected 34 of those claims.

*Regulatory efforts to collect data.* To develop data on “orphan” chemicals (those that were not sponsored in the voluntary portion of the HPV program and for which the U.S. EPA determined that data were still required), the agency began supplemental rule making under TSCA. Three TSCA Section 4 Test Rules were proposed, and later finalized (U.S. EPA 2006c, 2011g, 2011h), between December 2000 and October 2011. These rules required manufacturers to provide health and environmental effects data on 51 orphan chemicals that met Section 4 reporting criteria: *a*) the chemical is produced or enters the environment in substantial quantities or there is significant human exposure; *b*) existing data are inadequate for risk assessment; and *c*) testing is needed to develop the data required for the risk assessment (U.S. EPA 2011i). Comments supplied by APOs regarding these rules succeeded in eliminating or reducing animal testing for a number of chemicals [for examples, see Supplemental Material, pp. 2–3 (http://dx.doi.org/10.1289/ehp.1104666)]. In addition, the U.S. EPA issued TSCA Section 8(a) and Section 8(d) data reporting rules (U.S. EPA 2006a, 2006b) in August 2006 for 243 HPV chemicals, 35 of which were subsequently removed from the list of unsponsored substances subject to reporting under TSCA Section 8 (U.S. EPA 2006d, 2007).

On October 21, 2011, the U.S. EPA issued a proposal to collect data on 23 remaining HPV chemicals through a fourth and final TSCA Section 4 test rule (U.S. EPA 2011a). In the same notice, the agency also proposed to simultaneously issue a significant new use rule (SNUR) under TSCA Section 5(a)(2) for another 22 HPV chemicals. The SNUR would require manufacturers to file significant new use notifications (SNUN) with the U.S. EPA prior to any uses of the listed chemicals that would result in significant consumer or occupational exposure. This exercise of the U.S. EPA’s authority under TSCA Section 5 appears to allow the agency to effectively require new testing for HPV chemicals, including animal testing, without first finding that available data are insufficient to determine health or environmental effects, as required under TSCA Section 4. Such an approach would very likely lead to duplicative testing should the agency fail to comprehensively search for relevant data and provide opportunities for public review and comment. In addition, because companies producing the same chemical would likely cross the SNUN threshold at different times, and some may not cross it at all, the SNUR approach could lead to duplicative reporting requirements by defeating efforts to share costs and testing through formation of consortia.

*Outcomes and future of the HPV challenge program.* Sponsors were asked by the U.S. EPA to submit SIDS data no later than 2005 (U.S. EPA 2001); however, more than a decade since the start of the program, new test plans and revisions are occasionally submitted, and the U.S. EPA has posted information on its Robust Summaries and Test Plans web site (U.S. EPA 2012d) as recently as May 2012. The High Production Volume Information System (HPVIS), a web interface for accessing the hazard data (U.S. EPA 2012e), was not launched until April 2006, and efforts to familiarize potential users of the data have been limited to one national data-users conference held in December 2006 (U.S. EPA 2010b) and two regional workshops held in 2007 (U.S. EPA 2010c). Several methods of data query are offered on the HPVIS web site, but there is considerable variability in format and presentation of the data (e.g., multiple or inconsistent units), which limits the ability to use this information. Clearly, data formatting requirements should have been standardized early in the program.

The utility of the HPV data set for risk assessment is limited, acknowledged even by EDF, the organization that strongly advocated for the formation of the HPV Program, as “…provid[ing] little if any reliable, comprehensive information about the use of and exposure to HPV chemicals” (Denison 2007). The U.S. EPA has used some of the data in its now obsolete Chemical Assessment and Management Program to develop screening-level hazard, exposure, and risk characterizations for certain HPV chemicals (U.S. EPA 2012a). As part of its effort to identify and appropriately regulate chemicals of concern, the U.S. EPA has also produced action plans for 10 chemicals or groups of chemicals (U.S. EPA 2012b), 2 of which are produced in high volumes (bisphenol A and the nonylphenol/nonylphenol ethoxylates group), although identification of these substances as chemicals of concern does not appear to have been a direct result of data collection under the HPV program.

The voluntary nature of the HPV program and the limited data acquisition authority of the U.S. EPA under TSCA have led to a lengthy and fragmented data-gathering process, and attempts to update TSCA to address this problem have, thus far, been unsuccessful [see Supplemental Material (http://dx.doi.org/10.1289/ehp.1104666)]. Although it is appropriate to tailor data acquisition to meet regulatory needs, information also should be obtained in an organized, efficient manner.

In the 14 years since the HPV program began, numerous new methods, initiatives, and programs have been launched that promise to set priorities, reduce animal testing, and provide better regulation in the long run. Driven in part by the realization that animal testing is inefficient and that the information it provides is often difficult to use for regulatory purposes, the National Academy of Sciences published a seminal report, *Toxicity Testing in the Twenty-first Century: A Vision and a Strategy* (National Research Council 2007), which describes a novel and rational approach to chemical safety assessment and the reduction of whole animal testing. The U.S. EPA embraced this approach in its Strategic Plan for Evaluating the Toxicity of Chemicals (U.S. EPA 2009) and in the realignment and consolidation of several of its programs into the Chemical Safety for Sustainability Research Program (U.S. EPA 2011b). Furthermore, the U.S. EPA, the National Institutes of Health, and the FDA have invested heavily in Tox21, a collaboration to develop the technology necessary for this new approach (e.g., National Toxicology Program 2011). These strategies and tools are designed to provide more relevant information faster and less expensively than the current animal-based approach. They are already being incorporated into some of the U.S. EPA’s chemical safety programs as evidenced by the agency’s “Pesticide Program Vision for Enhancing Integrated Approaches to Testing and Assessment” (U.S. EPA 2011f), changes to its *Existing Chemicals Program: Strategy* (U.S. EPA 2012c), and the recent announcement of the *Endocrine Disruptor Screening Program for the 21st Century* (U.S. EPA 2011d, 2012g).

Considering the length of time it has been in existence and the increased number of chemicals that now meet the HPV definition, the HPV program as administered by the U.S. EPA has clearly been unable to keep pace with changes in the chemical industry. An industry-led initiative, Extended HPV (EHPV), was announced in 2005 (e.g., Sissell 2005) to expand the HPV program to include the 574 chemicals that had reached HPV levels since its start. Although some EHPV data have been submitted to the U.S. EPA, with the inception of the European Union’s mandatory Registration, Evaluation, Authorization and Restriction of Chemical Substances (REACh) regulation (European Commission 2006) in 2007, many global manufacturers and importers of chemicals have shifted their focus to developing data for REACh in the hope that the same data can be used to meet U.S. requirements. Notably, many of the chemicals included in the fourth TSCA Section 4 proposed test rule described above are either already registered under REACh or preregistered for the 31 May 2013 deadline. In comments on this test rule (ACC 2012), the chemical industry expressed concern over duplication of reporting requirements and called for the U.S. EPA to formally harmonize its test guidelines with those of the OECD, accept robust summaries of data submitted under REACh, and finalize a data-sharing agreement with the European Chemicals Agency (ECHA), which began with the signing of a Statement of Intent in December 2010 (ECHA 2010).

Compared with the threshold of 1 million pounds for the HPV program, REACh is decidedly more ambitious: Its goal is to comprehensively assess the safety of all chemicals produced or imported in Europe in quantities of ≥ 1 metric ton (2,205 pounds) (European Commission 2006). REACh prioritizes chemicals for testing by manufacture or import volume, and data requirements increase as the manufacture or import volume increases. The enabling legislation (European Commission 2006) contains language emphasizing the minimization of animal use and includes some measures corresponding to those implemented in the HPV program, such as grouping of chemicals and use of read-across. Moreover, a stated objective of the legislation is to promote non-animal test methods, and it provides a list of accepted alternative methods and other means of avoiding animal testing. However, a drawback to including specific testing methods is that it may be more difficult to adopt new methods as they become available, as exemplified by the current debate (e.g., ECHA 2011b) over the legality of replacing the two-generation reproductive toxicity test (OECD TG 416) with the new extended one-generation test (OECD TG 443) that reduces the number of animals used by half.

REACh guidance also includes detailed descriptions of integrated strategies that can be used to minimize testing and increase efficiency (e.g., ECHA 2008). As in the HPV program, actual efficiencies and reductions in animal use will depend on the degree to which these animal-saving measures are implemented. A recent preliminary assessment of the use of animal alternatives in the first phase of REACh (ECHA 2011a) indicated that formation of consortia by chemical companies greatly reduced duplicative animal testing and, as we observed for the HPV program, the use of existing data and read-across satisfied the largest number of end points requiring vertebrate animal testing. Not surprisingly, considering the current scarcity of universally accepted non-animal tests, data from only three *in vitro* methods—eye irritation, skin irritation, and genotoxicity—were submitted. This is likely to change as non-animal assessment tools, such as the (Q)SAR models collected in the OECD Toolbox (OECD 2010), continue to be developed and implemented.

## Conclusions

The U.S. HPV Program had the potential to consume > 3 million animals in health and environmental effects testing, but after involvement by APOs, a variety of animal-saving measures were introduced that reduced the number of animals actually used to approximately 127,000—still a considerable amount. Grouping related chemicals and applying read-across to estimate the toxicity of untested chemicals had the greatest impact on reducing animal use. Discovery of existing data by both APOs and chemical sponsors also substantially decreased testing on animals, a significant finding considering that the HPV program was founded on the premise that little hazard assessment data existed for HPV chemicals. Non-animal methods, such as *in vitro* tests and computer simulation, had comparatively little impact on mitigating animal use, satisfying only about 1% of the end points potentially requiring animal test data.

Of the animal tests that were conducted, combined protocols that could assess multiple end points in a single test significantly reduced animal use. Sponsors proposed these much more often than separate tests, and the U.S. EPA, in its test plan reviews, went on to recommend them in place of nearly all the separate tests proposed.

Because participation in the HPV program was voluntary and HPV sponsors may have had other reasons for conducting tests, the U.S. EPA’s recommendations to eliminate tests or conduct tests involving fewer or no animals were not always followed, thus resulting in almost 9,000 more animals being used. On the other hand, the voluntary nature of the HPV program saved animals by allowing sponsors to decline to do additional testing recommended by the U.S. EPA, although this testing may still be required through regulatory means at some point in the future. Ultimately, the impact of the HPV program on animals could have been far greater if APOs and other members of the public had not succeeded in advancing basic animal welfare principles shortly after it began.

The HPV program’s primary goal of making chemical hazard information available to the public seemingly has been met by the posting of raw data on the U.S. EPA’s web site (U.S. EPA 2012f). However, the U.S. EPA significantly underestimated the amount of time necessary to complete the program, and its own use of the data to assess the hazards of HPV chemicals has, for the most part, not progressed beyond the screening level stage. Although the data can be retrieved digitally, albeit through a somewhat cumbersome web interface, the extent to which the public is using it to participate in environmental decision making is unknown. Also, the HPV program did not systematically address information requirements by standardizing data reporting.

Both the HPV and REACh programs showcase the need for applying a different approach to prioritize chemicals for further evaluation and articulating targeted data requirements to increase the efficiency of chemical risk assessment. The science of toxicology is evolving rapidly, and although only some of the tools being developed as part of the Tox21 collaboration have been adequately evaluated for use in risk assessment, they can and are being used for prioritization and screening purposes. Building on the means by which animal testing was reduced in the HPV program, along with the development of new technologies, will likely increase the efficiency and efficacy of chemical hazard and risk assessment and continue to decrease the use of animals in chemical safety testing. However, decision makers must ensure incorporation of animal welfare principles and evolving chemical assessment strategies into current and future regulatory efforts in order for this to occur.

## Supplemental Material

(20 KB) PDF
