# Supplementary material for: Animal Use and Lessons Learned in the U.S. High Production Volume Chemicals Challenge Program
Source: Environ Health Perspect. 2012 Oct 2;120(12):1631–9. doi: 10.1289/ehp.1104666 (PMC3548278; doi:10.1289/ehp.1104666)
Supplement: (20 KB) PDF [file ehp.1104666.s001.pdf]

SUPPLEMENTAL MATERIAL

Animal Use and Lessons Learned in the
U.S. High Production Volume Chemicals Challenge Program

Patricia L. Bishop[1], Joseph R. Manuppello[1], Catherine E. Willett[1], Jessica T. Sandler[1]

[1]People for the Ethical Treatment of Animals, Regulatory Testing Division, Norfolk, VA, 23510

Table of Contents

APO comments on the second and third proposed Section 4 Toxics Substances Control Act (TSCA) test rules that reduced animal testing 2

TSCA Updates 3

References..3

APO comments on the second and third proposed Section 4 TSCA (Toxics Substances Control Act) test rules that reduced animal testing.

Animal Protection Organizations (APOs) submitted comments to EPA regarding High Production Volume (HPV) chemicals proposed for testing under Section 4 TSCA, a number of which resulted in reduction of animal testing or elimination of the chemical entirely from the test rule. Several examples are given below.

1. In comments submitted to EPA (Manuppello et al. 2008) for the proposed TSCA Section 4 test rule on the second group of HPV chemicals (U.S. EPA 2008), APOs stated that reproductive and developmental toxicity testing was not needed for 2,4-hexadienoic acid, (E,E)- (also known as sorbic acid), as several multi-generation studies in mice and rats (e.g., Demaree et al. 1955; Gaunt et al., 1975) reported no adverse effects of sorbic acid on reproductive function or post-natal development. The APO comments additionally cited the Food and Drug Administration (FDA) as listing sorbic acid as Generally Recognized as Safe (GRAS) as a preservative for direct addition to food. In its response (U.S. EPA 2010a), EPA reviewed the cited studies and acknowledged there was no evidence suggesting the chemical was either a reproductive or developmental toxicant, but did not make reference to the GRAS classification, which, in all likelihood, should have excluded this chemical from testing altogether. The agency stated it "…is not finalizing the reproduction/developmental toxicity screening test", but maintained that data on aquatic toxicity would still have to be developed (EPA 2010a).

2. Also in comments on the second proposed test rule (Manuppello et al. 2008), APOs noted that information existed (e.g., Goldman et al. 1977; Sheik-Omar and Scheifer 1980; Lamb et al. 1997) on the reproductive and developmental effects of ethanedioic acid (also known as oxalic acid) from several studies, including data from the National Toxicology Program (NTP 1985), thus obviating the need for reproductive/developmental toxicity testing. EPA reviewed the NTP study and noted in its response that while it did "…not conform entirely to current OECD reproductive and developmental testing guidelines, upon further consideration, it is considered adequate to eliminate the need for further reproductive and developmental testing under a test rule" (U.S. EPA 2010a).

3. The third proposed test rule (U.S. EPA 2010b) required mammalian acute toxicity and combined repeated-dose reproduction/developmental toxicity screening tests for Benzene, 1,2-dimethyl-3-nitro- (3-NOX). In comments (Manuppello 2010), APOs referenced an abstract (Anonymous 1994) from a 1994 German toxicology assessment in the TOXLINE database (U.S. NLM 2011) that summarized results of oral acute and repeated dose toxicity studies of 3-NOX in rats. Upon examination of the studies, EPA determined that they provided sufficient information to adequately characterize the acute and repeated dose hazards of oral exposure to 3-NOX (U.S. EPA 2011).

4. EPA called for reproductive/developmental toxicity testing of 3-pentanone in its third proposed test rule (U.S. EPA 2010b). With regards to 3-pentanone, APOs urged EPA in their comments (Manuppello 2010) to consider the existing data for 2-pentanone, a structurally similar chemical that was already sponsored in the HPV Challenge program and for which all health effects endpoints were fulfilled. EPA stated in its response (U.S.

EPA 2011) that it assessed the existing data for 2-pentanone, and based on similar chemical/physical properties and comparative acute lethality data, considered a read-across approach from 2-pentanone to 3-pentanone to be appropriate. EPA further stated that toxicity data submitted for 2-pentanone were adequate to satisfy all testing requirements for 3-pentanone, and it would drop the chemical from the test rule (U.S. EPA 2011).

TSCA Updates

Bills that would significantly rewrite TSCA have been introduced three times since 2008 (Kid Safe Chemicals Act of 2008; Safe Chemicals Act of 2010; Safe Chemicals Act of 2011) and though none have received much support in Congress, each would change the legislation to look more like the European REACh (Registration, Evaluation, Authorization and Restriction of Chemical Substances) Program (European Commission 2006) by requiring information on more chemicals and toxicological endpoints. The latest versions contain language that promotes minimization of animal testing and supports development of non-animal assessment approaches, but other elements of these drafts are still contentious and subject to further discussion and amendment. By the time TSCA legislation is revised in the U.S., information for at least the first two categories of REACh likely will have been generated, as the deadline for substances produced or imported at greater than 100 tonnes per year is December 2013 (European Commission 2006). Any revision of TSCA should take this into account and allow use of this information to the greatest extent possible.

REFERENCES

Anonymous. 1994. Toxikologische Bewertung. Berufsgenossenschaft der chemischen Industrie. Heidelberg 93: 16.

Demaree, GE, Sjogren, DW, McCashland, BW, and Cosgrove, EP. 1955. Preliminary studies on the effect of feeding sorbic acid upon the growth, reproduction and cellular metabolism of albino rats. J. Amer Pharm Assoc 44: 619-621.

European Commission. 2006. Regulation (EC) No 1907/2006 of the European Parliament and of the Council of 18 December 2006. Off J Euro Union L396:1-849. Available: http://eurlex.europa.eu/LexUriServ/LexUriServ.do?uri=oj:l:2006:396:0001:0849:en:pdf [accessed 22 June 2012]

Gaunt, IF, Butterworth, KR, and Gangolli, SD. 1975. Long-term toxicity of sorbic acid in the rat. Food and Cosmetics Tox 13: 31-45.

Goldman M, Doering GJ, Nelson RG. 1977. Effect of dietary ingestion of oxalic acid on growth and reproduction in male and female Long-Evans rats. Research Commun Chem Pathol Pharmacol 18:369-72.

Kid Safe Chemicals Act of 2008, S. 3040. 2008. 110th Cong., 2nd Sess. Available: http://www.govtrack.us/congress/bills/110/s3040/text [accessed 22 June 2012].

Lamb, JC IV, et al. 1997. Oxalic Acid. Env Health Persp 105 (Suppl 1): 229-230.

Manuppello, J, Sullivan, K, and Beck, N. 2008. Comments on "Testing of Certain High Production Volume Chemicals; Second Group of Chemicals"submitted on behalf of People for the Ethical Treatment of Animals (PETA), the Physicians Committee for Responsible Medicine (PCRM), the Alternatives Research Development Foundation, and the American Anti-Vivisection Society. Available: http://www.regulations.gov/#!documentDetail;D=EPA-HQ-OPPT-2007-0531-0064 [accessed 22 June 2012].

Manuppello, J. 2010. Comments on "Testing of Certain High Production Volume Chemicals; Third Group of Chemicals" submitted on behalf of People for the Ethical Treatment of Animals (PETA), the Physicians Committee for Responsible Medicine (PCRM), the Alternatives Research Development Foundation, and the American Anti-Vivisection Society. Available: http://www.regulations.gov/#!documentDetail;D=EPA-HQ-OPPT-2009-0112-0069 [accessed 22 June 22, 2012].

NTP (National Toxicology Program). 1985. Oxalic acid (CAS No. 144-62-7). Reproduction and fertility assessment in CD-1 mice when administered in drinking water. Abstract, NTP Report No. RACB84064.

Safe Chemicals Act of 2010, S. 3209. 2010. 111th Cong., 2d Sess. Available: http://www.govtrack.us/congress/bills/111/s3209/text [accessed 22 June 2012].

Safe Chemicals Act of 2011, S. 847. 2011. 112th Cong., 1st Sess. Available: http://lautenberg.senate.gov/assets/SafeChem.pdf [accessed 22 June 2012].

Sheik-Omar AR and Schiefer HB. 1980. Effects of feeding oxalic acid to pregnant rats. Pertanika. 3:25-31.

U.S. EPA (U.S. Environmental Protection Agency). 2008. Testing of Certain High Production Volume Chemicals-Second Group of Chemicals; Proposed Rule. Fed. Reg. 73: 43314-43342.

U.S. EPA (U.S. Environmental Protection Agency). 2010a. Testing of Certain High Production Volume Chemicals; Second Group of Chemicals; Final Rule, Response to Public Comments, November 22, 2010. Available: http://www.regulations.gov/#!documentDetail;D=EPA-HQ-OPPT-2007-0531-0580 [accessed 22 June 2012].

U.S. EPA (U.S. Environmental Protection Agency). 2010b. Testing of Certain High Production Volume Chemicals; Third Group of Chemicals; Proposed Rule. Fed. Reg. 75: 8575-8601.

U.S. EPA (U.S. Environmental Protection Agency). 2011. Testing of Certain High Production Volume Chemicals; Third Group of Chemicals; Final Rule Response to Public Comments,

September 12, 2011. Available: http://www.regulations.gov/#!documentDetail;D=EPA-HQ-OPPT-2009-0112-0080 [accessed 22 June 2012]

U.S. NLM (National Library of Medicine). 2011. Toxicology Literature Online. Available: http://toxnet.nlm.nih.gov/cgi-bin/sis/htmlgen?TOXLINE [accessed 22 June 2012].
